# Residual normal B-cell profiles in monoclonal B-cell lymphocytosis versus chronic lymphocytic leukemia

**DOI:** 10.1038/s41375-018-0164-3

**Published:** 2018-06-21

**Authors:** Ignacio Criado, Elena Blanco, Arancha Rodríguez-Caballero, Miguel Alcoceba, Teresa Contreras, María Laura Gutiérrez, Alfonso Romero, Paulino Fernández-Navarro, Marcos González, Fernando Solano, Carlos Gómez, Martín Pérez-Andrés, Jacques J. M. van Dongen, Julia Almeida, Alberto Orfao, EuroFlow PID Group and The Primary Health Care Group of Salamanca for the Study of MBL

**Affiliations:** 1 Cancer Research Center (IBMCC, USAL-CSIC), Department of Medicine and Cytometry Service (NUCLEUS), University of Salamanca, CIBERONC and IBSAL, Salamanca, Spain; 2 Hematology Service, University Hospital of Salamanca, IBMCC, CIBERONC, IBSAL and Department of Nursery and Physiotherapy, University of Salamanca, Salamanca, Spain; 3 Biochemistry Service, University Hospital of Salamanca, Salamanca, Spain; 4 Centro de Atención Primaria de Salud Miguel Armijo, Salamanca, Sanidad de Castilla y León (SACYL), Castilla y León, Spain; 5 Centro de Atención Primaria de Salud de Ledesma, Salamanca, Sanidad de Castilla y León (SACYL), Castilla y León, Spain; 6 Hematology Service, Hospital Nuestra Señora del Prado, Talavera de la Reina, Toledo, Spain; 7 Department of Immunohematology and Blood Transfusion, Leiden University Medical Center, Leiden, The Netherlands

Chronic lymphocytic leukemia (CLL) is the most common adult leukemia in Western countries, which is characterized by the accumulation of mature CD5^+^/CD20^lo^/CD23^+^ clonal B-cells in peripheral blood (PB), bone marrow (BM), and other lymphoid tissues [1]. Currently, it is well-established that CLL is systematically preceded by a pre-leukemic stage, known as monoclonal B-cell lymphocytosis (MBL) [2]; MBL includes both low-count (MBL^lo^) and high-count MBL (MBL^hi^), depending on the number of PB clonal B-cells (<0.5 × 10^9^/L and ≥0.5 × 10^9^/L, respectively) detected [3], the former being a highly prevalent condition in adults (≈25% of individuals >70 y) [4, 5]. The biological and clinical significance of CLL-like clonal B-cells in PB of otherwise healthy individuals (MBL^lo^) has not been fully elucidated [6–8]. Recently, we have reported a very low rate of transformation of MBL^lo^ to MBL^hi^/CLL, after 7 years of follow-up [8]. In contrast, we found a higher frequency of deaths in MBL^lo^ subjects vs. age- and sex-matched non-MBL healthy adults from the same geographical area; among the former subjects, infection was an overrepresented cause of death (21% vs. 2%, respectively) [8]. This is in line with previous studies showing an ≈3-fold increased risk of infection in both MBL^hi^ and CLL patients, in whom infections also represent a major cause of death [9, 10].

Altogether, the above findings suggest an impaired immune system and immune surveillance, already at very early CLL stages. So far, several immunological defects of both the innate and adaptive compartments of the immune system have been reported in CLL, including hypogammaglobulinemia and an impaired T- and NK-cell function [10]. However, the precise mechanisms that lead to this CLL-associated secondary immunodeficiency state still remain poorly understood, and little is known about the specific (pre-leukemic) stage of onset of the impaired immune response. Since hypogammaglobulinemia is one of the most common and relevant alterations involved in the secondary immunodeficiency of most CLL patients, here we investigated the composition of the residual normal PB B-cell compartment in both MBL^lo^ and MBL^hi^ vs. early (Rai stage 0) CLL, to gain insight into the mechanisms involved in hypogammaglobulinemia in CLL, and the precise stage at which the first alterations occur.

Overall, 110 subjects—61 males (55%) and 49 females (45%); mean age: 72 ± 11 y—were prospectively enrolled in this study between January 2015 and June 2017, with no seasonal differences in recruitment for the distinct groups analyzed. Subjects were classified into: controls (40 non-MBL^lo^ healthy adults), MBL^lo^ (*n* = 27), MBL^hi^ (*n* = 21), and CLL stage 0 (CLL-0) patients (*n* = 22). Identification and characterization of residual normal PB B-cells and quantitation of immunoglobulin (Ig) levels was performed using high-sensitivity flow cytometry and nephelometry/turbidimetry, respectively. Inclusion criteria, flow cytometry protocols, panels and reagents, as well as the immunophenotypic criteria used for the identification of the different PB B-cell subsets, together with the clinical and biological characteristics of all individuals analyzed, are detailed in Supplementary Methods, Supplementary Tables 1–4, and Supplementary Figure 1.

Overall, both MBL^hi^ and CLL-0 patients showed significantly reduced normal PB B-cell counts (Fig. 1a), at the expense of pre-germinal center (GC) (immature and naïve) B-cells (*P* ≤ 0.001; Fig. 1b), while no significant differences were observed in MBL and CLL-0 vs. non-MBL controls regarding total PB memory B cells (MBC) (Fig. 1c). In turn, the overall PB plasma cell (PC) compartment was significantly reduced (vs. controls) among MBL^hi^ subjects (*P* = 0.002), but not in CLL and MBL^lo^ cases (Fig. 1c). These results confirm and extend on previous findings from our group showing that production and release of both immature and naïve B-cells into PB is already reduced in MBL [11]. Currently, it is well-established that during adulthood, PB MBC and PC counts (but neither PB immature nor naïve B-cell numbers) progressively decrease with age [12]; therefore, age alone could not explain the lower pre-GC B-cell counts reported here among MBL^hi^ and CLL-0 cases, also because a similar age distribution was observed among all groups analyzed (Supplementary Table 4; Supplementary Figure 2). Conversely, the decreased numbers of pre-GC B-cells in PB of MBL^hi^ subjects suggests an impaired production of (newly generated) B-cells in the BM, already at the earliest disease stages. This might be due to a decreased number of available BM niches, as soon as they are (progressively) occupied by CD5^+^ CLL-like clonal B-cells. Thus, previous studies have suggested that BM infiltration by CLL cells displaces other resident cell populations (e.g., normal B-cell precursors), and generates an impaired hematopoietic microenvironment [13]. Interestingly, BM infiltration at early disease stages might preferentially affect the B-cell niches, since (by definition) no other cytopenias were observed in MBL and CLL-0 patients. BM analyses would then become crucial to better understand the underlying B-cell depletion mechanisms in these subjects; due to ethical reasons and the lack of medical indication for BM sampling in MBL, BM samples were not collected here. However, if the above hypothesis holds true, decreased BM production of B-lymphocytes, in the transition from MBL^lo^ to MBL^hi^ and CLL, would probably translate into a progressively narrower B-cell repertoire and progressively lower coverage of all required antigen specificities and, thereby, to defective (new) B-cell responses against specific pathogens, as recently reported for pneumococcus [14]. Further *IGH* repertoire analyses of purified normal pre-GC B-cell subsets from both MBL and CLL subjects are required to fully confirm this hypothesis.

**Fig. 1:**
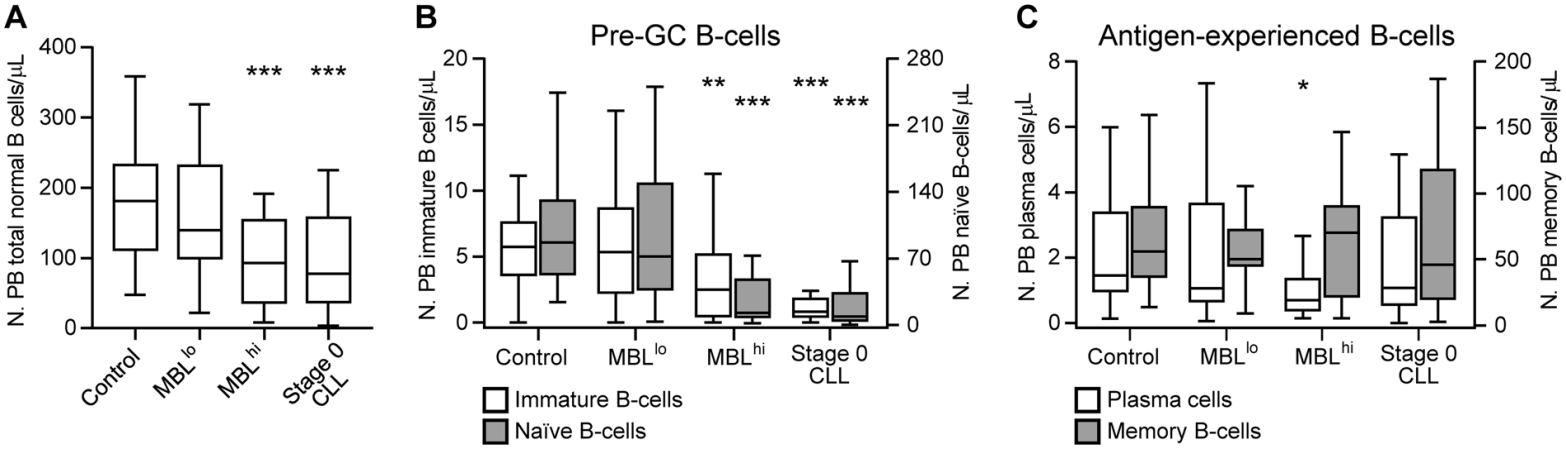
Distribution of normal residual B-cells and their major subsets in peripheral blood of MBL and CLL cases vs. non-MBL controls. **a** The absolute number of residual normal B-cells. **b** The absolute number of pre-germinal center B-cells; white boxes represent immature B-cells (left *Y*-axis scale), while gray boxes represent naïve B-cells (right *Y*-axis scale). **c** The absolute number of antigen-experienced B-cells; white boxes represent plasma cells (left *Y*-axis scale), while gray boxes represent memory B-cells (right *Y-*axis scale). In all panels, notched boxes represent 25th and 75th percentile values; the lines in the middle correspond to median values and vertical lines represent the highest and lowest values that are neither outliers nor extreme values. *P ≤ 0.05 vs. controls; **P ≤ 0.01 vs. controls; ***P ≤ 0.001 vs. controls. MBL^lo^ low-count monoclonal B-cell lymphocytosis, MBL^hi^ high-count monoclonal B-cell lymphocytosis, CLL chronic lymphocytic leukemia

Although total PB PC numbers were only reduced in MBL^hi^ and no statistically significant differences were observed in total MBC counts among the groups here studied, an altered distribution of B-cell subsets expressing distinct Ig-subclasses was observed among both antigen-experienced B-cell populations in MBL and CLL (Fig. 2). Such altered distribution was progressively more marked from MBL^lo^ to MBL^hi^ and CLL-0. Thus, while in MBL^lo^ only slightly decreased IgM^+^ PC counts were found in the PB (*P* = 0.05; Supplementary Figure 2A), together with normal MBC and Ig levels (Fig. 2c,d; Supplementary Figure 4 and Supplementary Figure 5), MBL^hi^ subjects showed reduced numbers of PC populations of all Ig-subclass (Fig. 2a,b), except IgG3^+^ PC (Supplementary Figure 3B), together with lower numbers of IgG3^+^ and IgG4^+^ MBC (Supplementary Figure 4B and 4F). In turn, CLL-0 patients showed decreased IgM^+^, IgG2^+^, IgG4^+^, and IgA2^+^ PC counts (Supplementary Figure 3) and low IgG2^+^, IgG4^+^, and IgA2^+^ MBC numbers (Supplementary Figure 4), which translates into overall decreased numbers of PCs and MBCs expressing those Ig-subclasses encoded downstream in the *IGHC* gene (Fig. 2b). Of note, no seasonal differences existed in recruitment among the four study groups, suggesting that differences in PC and MBC subset numbers were not influenced by seasonal changes.

**Fig. 2:**
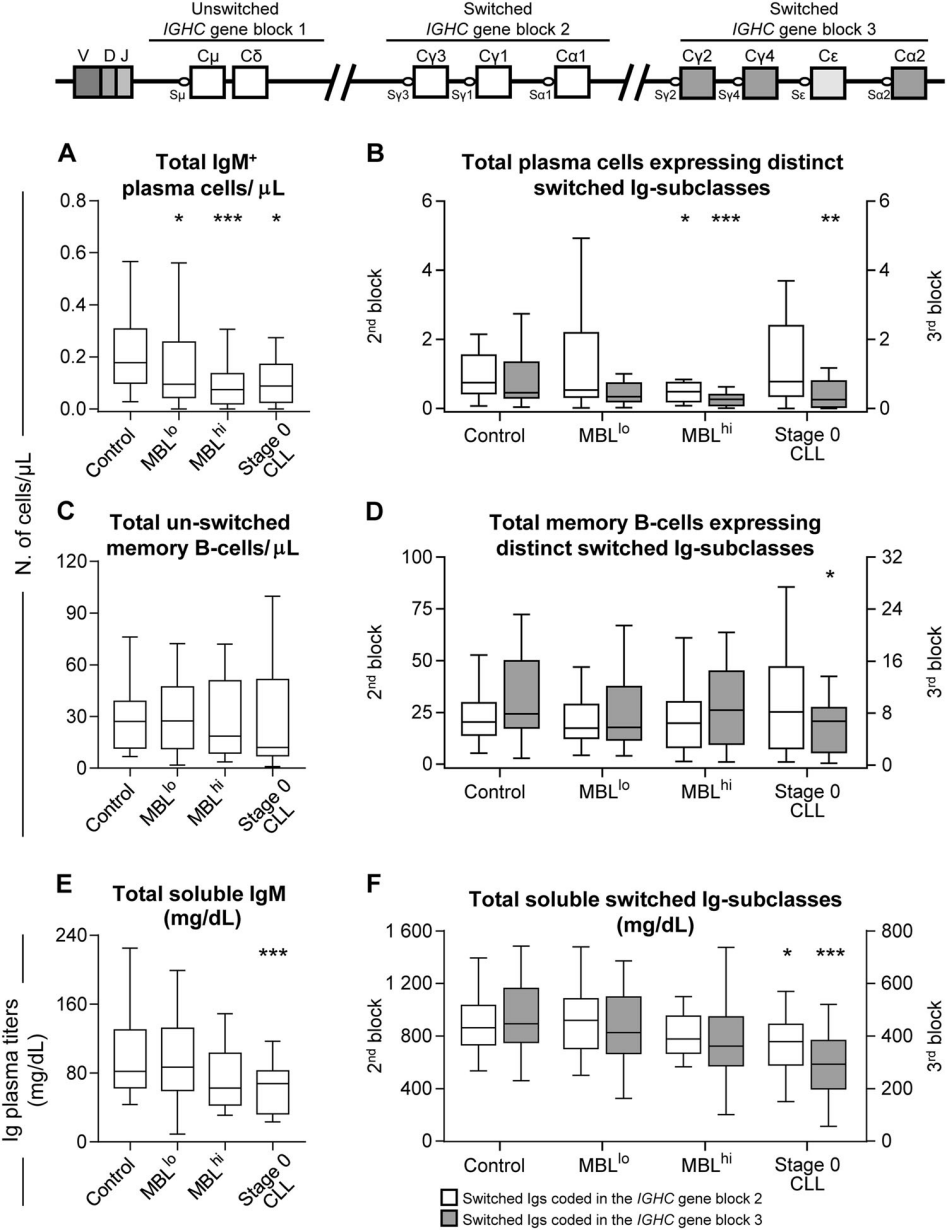
Distribution of PB antigen-experienced B-cell subsets expressing distinct Ig-subclasses and soluble Ig-subclass plasma titers grouped according to the position they occupy in the *IGHC* gene blocks. **a**, **b** The absolute number of IgM^+^ and switched plasma cells, respectively. **c**, **d** The absolute number of IgMD^+^ un-switched memory B-cells and switched memory B-cells are displayed, respectively. **e**, **f** Soluble IgM titers in plasma and the sum of the soluble levels of the different switched Ig-subclasses according to the distinct position that they occupy in the *IGHC* gene, respectively. **b**, **c**, and **f** White boxes represent the sum of those Ig-subclasses encoded in the second *IGHC* gene block, while gray boxes represent the sum of those Ig-subclasses encoded in the third *IGHC* gene block. The relative position and order of the different gene segments of the *IGHC* gene that encode for the different Ig-subclasses are depicted on the top of the figure. Notched boxes represent 25th and 75th percentile values; the lines in the middle correspond to median values and vertical lines represent the highest and lowest values that are neither outliers nor extreme values; *P ≤ 0.05 vs. controls; **P ≤ 0.01 vs. controls; ***P ≤ 0.001 vs. controls. MBL^lo^ low-count monoclonal B-cell lymphocytosis, MBL^hi^ high-count monoclonal B-cell lymphocytosis, CLL chronic lymphocytic leukemia

Regarding plasma Ig titers, soluble IgM levels were significantly reduced in both MBL^hi^ (*P* = 0.03) and CLL-0 (*P* = 0.008) (Fig. 2e); in addition, MBL^hi^ showed reduced IgG2 and IgG4 soluble levels (Supplementary Figure 5C-E) while CLL-0 patients displayed overall decreased plasma levels of all IgG-subclasses (*P* ≤ 0.02), particularly also of those encoded downstream in the *IGHC* gene (i.e., IgG2, IgG4, and IgA2; *P* ≤ 0.001; Fig. 2f). Thus, the overall reduction in soluble IgG titers was mostly at the expense of Ig-subclasses coded downstream in the third block *IGHC* gene, mimicking the altered PC and MBC profiles described above for the same patients (Supplementary Figure 5).

Altogether, these results suggest that IgM^+^ PC responses are already hampered in MBL^lo^, while they are associated with different patterns of alteration of other normal residual antigen-experienced B-cells in MBL^hi^ and CLL-0. Thus, while in MBL^hi^ almost all PC populations were already reduced, and only few (decreased IgG3^+^ and IgG4^+^ MBC) alterations were observed in the distribution of the distinct MBC subpopulations analyzed, a lower number of PC subsets, together with a greater number of MBC subsets, were affected in CLL-0. Of note, MBC and PC expressing Ig-subclasses which are encoded downstream in the *IGHC* gene (i.e., IgG2^+^, IgG4^+^, and IgA2^+^) were the only antigen-experienced B-cell populations decreased in CLL (Fig. 2a, d).

These later findings point out the existence of a progressive deterioration of B-cell responses driven by newly encountered Ags from MBL^lo^ to MBL^hi^ and CLL-0. This is likely due to an impaired pre-GC B-cell production, that would lead to a progressively reduced B-cell repertoire, with decreased production of new Ag-experienced B-cells from MBL^lo^ to MBL^hi^ and CLL-0. This immunodeficiency state might explain the previously reported reactivation in CLL of B-cell responses against common pathogens, particularly host-viruses such as cytomegalovirus (CMV) and Epstein Barr virus (EBV) [14]. The regeneration of PB PC numbers here reported between MBL^hi^ and CLL-0 could be, thereby, due to such reactivation of antibody responses against common (dominant) antigens, including new antibody responses against CMV and EBV [14]; this is consistent with the apparent recovery of the number of PB PCs (and also MBCs) expressing Ig-subclasses, which are coded upstream in the *IGHC* gene block (i.e., IgG3^+^, IgG1^+^, and IgA1^+^) as found here for CLL-0 patients. These results would also support the higher frequency of infections driven by encapsulated bacteria in MBL and CLL patients, since IgG2 is the main actor in the humoral defense against polysaccharide antigens, and it was significantly reduced in both MBL^hi^ and CLL [15]. Further longitudinal long-term follow-up studies in larger series of newly diagnosed/untreated MBL and CLL patients, including functional antigen-specific PC and MBC in vitro assays, are necessary to confirm this hypothesis.

## Electronic supplementary material

Supplementary Information
