# Supplementary material for: Residual normal B-cell profiles in monoclonal B-cell lymphocytosis versus chronic lymphocytic leukemia
Source: Leukemia. 2018 Jun 21;32(12):2701–5. doi: 10.1038/s41375-018-0164-3 (PMC6286325; doi:10.1038/s41375-018-0164-3)
Supplement: Supplementary file 1 — Supplementary Information [file 41375_2018_164_MOESM1_ESM.pdf]

SUPPLEMENTARY INFORMATION

Supplementary Material and Methods:

Subjects and samples. A total of 110 subjects –61 males (55%) and 49 females (45%); mean age of 72±11y– were prospectively enrolled in this study between January 2015 and June 2017. Subjects were classified into: controls (40 non-MBL^{lo} adult healthy donors), low-count monoclonal B-cell lymphocytosis –MBL^{lo}– (n=27), high-count monoclonal B-cell lymphocytosis –MBL^{hi}– (n=21) and Rai stage 0 chronic lymphocytic leukemia –CLL-0– patients (n=22). Controls and MBL^{lo} subjects were recruited at the Primary Health Care Service of Salamanca (Salamanca, Spain), while MBL^{hi} and untreated/newly-diagnosed CLL-0 patients were recruited at the Service of Hematology of the University Hospital of Salamanca (Salamanca, Spain) and the *Nuestra Señora del Prado* Hospital (Toledo, Spain). According to the WHO 2017 criteria, diagnosis of CLL-like MBL was established whenever $<5x10^{9}$ peripheral blood (PB) clonal B-cells/L with a CLL-like immunophenotype were detected, in the absence of any other CLL-associated symptoms or signs of disease.[1] MBL was further subdivided into MBL^{lo} ($<0.5x10^{9}$ CLL-like clonal B-cells/L) and MBL^{hi} ($\geq 0.5x10^{9}$ CLL-like clonal B-cells/L).[1] In turn, diagnosis of CLL was made following the WHO-2017 and the International Workshop on CLL criteria.[1,2]

Inclusion criteria for controls were as follows: i) age >40y, ii) absence of recent infectious diseases, and iii) no current or past history of autoimmune diseases or any immunomodulatory treatment (e.g. corticosteroids, chemotherapy), including administration of intravenous immunoglobulins. All subjects gave their written informed consent to participate, and the study was approved by the Ethics Committee of the University Hospital of Salamanca/IBSAL.

Flowcytometry immunophenotypic studies. Between 1-2 ml EDTA-anticoagulated PB were collected/case and within 24h, lysed using ammonium chloride and stained with a panel of fluorochrome-conjugated antibodies, following well-established protocols[3,4] and previously validated antibody combinations (Supplementary Table 1).[5] These combinations of monoclonal antibodies allowed identification of CLL-like and CLL clonal cells as well as the main normal PB B-cell populations (i.e. immature, naïve, plasma cells and memory B cells), including the subsets of antigen-experienced B-cells expressing different Ig isotypes and subclasses according to their

distinct phenotypes as described elsewhere,[5–7] and detailed in Supplementary Table 2. For each sample, ≥5x10^6 total PB leukocytes were measured in a FACSCanto II (8-color panel) or an LSR Fortessa X-20 (14-color panel) flow cytometer –Becton/Dickinson Biosciences (BD), San José, CA–. Instrument setup, calibration and daily quality control were performed following the EuroFlow standard operating procedures (SOP) available at www.EuroFlow.org. For data analysis the INFINICYT™ V2.0 software (Cytognos, Salamanca, Spain) was used.

Interphase fluorescence *in situ* hybridization (iFISH) and *IGHV* mutational status analyses. Fluorescence-activated cell sorting (FACS)-sorted (≥95% purity) clonal B-cell populations were investigated for the presence of CLL-related cytogenetic alterations (Supplementary Table 3) –i.e. del(13q14)(*D13S25*), trisomy 12, del(11q)(*ATM*) and del(17p)(*TP53*)–, and their *IGHV* gene rearrangements and mutational status, as described elsewhere.[8–10] The IMGT database (www.imgt/cines.org) was used to analyze the *IGHV* sequences obtained; sequences with an *IGHV* germline identity <98% were considered to be mutated (M), while those with ≥98% germline identity were classified as unmutated (UM).[11,12]

Quantitation of plasma soluble Ig isotype-subclass levels. Soluble IgM and IgG_{1-4} subclass levels were evaluated in platelet-depleted plasma samples using conventional nephelometry –Dimension Vista® (Siemens Healthcare, Erlanger, Germany)–, and IgA_{1-2} levels were assessed using the SPAPLUS® turbidimetric system (Binding Site, Birmingham, UK); for both approaches, the manufacturers SOPs and recommendations were strictly followed.

Statistical analyses. The Mann-Whitney U and Chi-square tests were used to establish the statistical significance of differences observed between groups for continuous and categorical variables, respectively. *P*-values ≤0.05 were considered to be associated with statistical significance. All statistical analyses were performed using the SPSS V19.0 software (SPSS-IBM, Armonk, NY).

Supplementary Tables

SUPPLEMENTARY TABLE 1. Combinations of fluorochrome-conjugated antibodies used for flow cytometric immunophenotypic identification and characterization of PB B-cell subsets in each subject included in this study.

LST (EuroFlow® Lymphocyte Screening Tube):
8-color tube acquired in a FACSCanto II (BD) flow cytometer used for the identification of the major leukocyte populations in PB

Fluorochrome	PacB	OC515	FITC	PE	PerCPCy5.5	PECy7	APC	APC-H7
**Marker**	CD20 + CD4	CD45	CD8 + Anti-κ	CD56 + Anti-λ	CD5	CD19 + anti-TCRγδ	CD3	CD38
**Clone**	2H7 + RPA-T4	HI30	UCHT-4 + Polyclonal	C5.9 + Polyclonal	HIB19	HI10a + 11F2	UCHT2	HB7
**Source**	eBioscience + BD	Cytognos	Cytognos	Cytognos	BD	Beckman Coulter + BD	BD	BD

Abbreviations (alphabetical order): **APC:** allophycocyanine; **APCH7:** allophycocyanine-hilite®7; **BD:** Becton/Dickinson Biosciences; **FITC:** fluorescein isothiocyanate; **PacB:** pacific blue™; **PE:** phycoerythrin; **PECy7:** phycoerythrin-cyanine7; **PerCPCy5.5:** peridinin chlorophyll protein cyanine 5.5; **OC515:** Orange Cytognos 515.

EuroFlow® Ig isotype subclass B-cell tube:
12-color tube acquired in an LSR Fortessa X-20 (BD) instrument and used for the identification of the different subsets of normal residual PB B-cells including those defined by the expression of distinct Ig-subclasses

Fluorochrome	BV421	BV510	BV605	BV711	BV786	FITC	PerCP-Cy5.5	PE	PE-CF594	PECy7	APC	APC-H7
**Marker**	CD27	smIgM	CD5	CD21	CD19	smIgG3 + smIgG2	smIgA1 + smIgA2	smIgG1 + smIgG2	smIgD	CD20	smIgG4 + smIgA1	CD38
**Clone**	M-T271	MHM-88	UCHT2	B-ly4	SJ25C1	SAG3 + SAG2	SAA1 + SAA2	SAG1 + SAG2	IA6-2	2H7	SAA1 + SAG4	HB7
**Source**	BD	Biolegend	BD	BD	BD	Cytognos	Cytognos	Cytognos	BD	BD	Cytognos	BD

Abbreviations (alphabetical order): **APC:** allophycocyanine; **APCH**7: allophycocyanine-hilite®7; **BD:** Becton/Dickinson Biosciences; **BV421:** brilliant violet 421; **BV510:** brilliant violet 510; **BV605:** brilliant violet 605; **BV711:** brilliant violet 711; **BV786:** brilliant violet 786; **FITC:** fluorescein isothiocyanate; **PE:** phycoerythrin; **PE-CF594:** phycoerythrin-carboxyfluorescein594; **PECy7:** phycoerythrin-cyanine7; **PerCPCy5.5:** peridinin chlorophyll protein cyanine 5.5.

SUPPLEMENTARY TABLE 2. Phenotypic profiles and criteria used for the identification of the different PB circulating B-cell subsets.

	PB B-cell and PC populations					
**Phenotypic markers**	**Immature B-cells**	**Naïve B-cells**	**Un-switched Memory B-cells**	**Switched Memory B-cells**	**Un-switched Plasma cells**	**Switched Plasma cells**
CD5	+	+/-	-	-	-	-
CD19	+	+	+	+	$+^{lo}$	$+^{lo}$
CD20	+	+	+	+	$+^{lo}$	$+^{lo}$
CD21	+	+/-	+/-	+/-	-	-
CD27	-	-	+	+/-	++	++
CD38	+	-	-	-	++	++
sIgM	++	+	+	-	$+^{lo}$	-
sIgD	+	++	+	-	-	-
$sIgG_{1-4}$ or $sIgA_{1-2}$	-	-	-	+#	-	+#

[#]Each switched memory B-cell or plasma cell only expresses one of the possible IgG or IgA subclasses at a time. Immunophenotypes in this table were defined according to Sims et al., Agrawal et al and Blanco et al.[13–15]

SUPPLEMENTARY TABLE 3. Panel of fluorochrome-conjugated probes used for interphase fluorescence in situ hybridization (iFISH) studies and the corresponding chromosomal regions targeted.

**Probe name**	**Fluorophore**	**Targeted chromosome band/ region**	**Probe size (Kb)**#
Custom Kit for CLL-like clones in MBL^{lo}.			
ATM	SG	11q22.3	180
D12Z3	SA	12p11.1-q11	NA
D13S25	SGo	13q14.3	306
P53	SR	17p13.1	159
LSI IGH DC BA	SO/SG	14q32	250/900
Probes for MBL^{hi} and CLL			
LSI ATM	SO	11q22	230
D12Z3	SO	12p11.1-q11	NA
LSI D13S25	SO	13q14.3	160
LSI TP53	SO	17p13	145
LSI IGH DC BA	*SO/SG*	*14q32*	*250/900*

#Information obtained from Kreatech Diagnostics (Amsterdam, The Netherlands), CytoCell Ltd. (Cambridge, UK) and Vysis Inc. (Abbott Park, IL). Customized probes kits from CytoCell Ltd. Abbreviations (alphabetical order): **BA:** break apart; **LSI:** locus specific identifier; **NA:** not applicable; **SA:** spectrum aqua; **SG:** spectrum green; **SGo:** spectrum gold; **SO:** spectrum orange; **SR:** spectrum red.

SUPPLEMENTARY TABLE 4. Clinical and biological characteristics of MBL and CLL subjects vs. non-MBL healthy controls.

	**Controls (n=40)**	**MBL^{lo} (n=27)**	**MBL^{hi} (n=21)**	**CLL Rai stage 0 (n=22)**	***P-value***
Male / Female#	18 / 22 (45%/55%)	14 / 13 (52%/48%)	16 / 5 (76%/24%)	13 / 9 (59%/41%)	0.02[b]
Age (years)	71 (52-96)	74 (52-91)	75 (58-92)	72 (52-89)	NS
Neutropenia# (<1 000/µL)	0 (0%)	0 (0%)	0 (0%)	0 (0%)	NS
N. leukocytes /µL	7 050 (4 340-10 390)	6 820 (3 010-11 630)	11 120 (6 400-19 300)	24 560 (10 050-85 740)	<0.001[b,d,c,e,f]
N. lymphocytes /µL	2 341 (938-4 057)	2 135 (621-5 103)	5 433 (1 910-7 576)	19 533 (7 590-77 126)	0.01[d,c,e,f]
N. total T cells /µL	1 693 (636-3 130)	1 592 (460-3 018)	1 688 (744-3 048)	2 045 (1 042-5 349)	0.02[e]
N. $CD4^+$ T cells /µL	1 067 (345-1 615)	835 (227-2 045)	894 (584-2 179)	1 193 (522-3 426)	0.03[e]
N. $CD8^+$ T cells /µL	520 (146-1 653)	528 (182-1 742)	498 (55-1 495)	729 (178-1 577)	NS
N. $CD4^+$/$CD8^+$ T cells /µL	7.0 (0-156)	6.7 (1.5-23)	8.5 (0.62-79)	11 (2.4-306)	0.03[e]
N. $CD4^-$/$CD8^-$ T cells /µL	80 (4.2-354)	66 (16-254)	61 (1.7-344)	107 (19-461)	0.04[c,f]
N. NK cells /µL	345 (135-1 273)	428 (89-1 854)	303 (31-1 157)	453 (68-1 030)	NS
N. total B cells /µL	182 (48-359)	146 (26-614)	2 675 (691-4 610)	16 040 (5 241-73 014)	<0.001[b,c,e,f]
N. normal B cells /µL	182 (48-359)	140 (21-480)	93 (8.2-740)	78 (3.7-1 022)	<0.001[b,d,c,e,f]
N. clonal B cells /µL	NA	1.7 (0.04-326)	2 529 (509-4 602)	15 964 (5 207-72 673)	<0.02[d,e,f]
IGHV mutational status# (M / UM)	NA	9 / 2 (82%/18%)	19 / 0 (100%/0%)	15 / 6 (71%/29%)	<0.05[d,f]
Cytogenetic alterations					
TOTAL altered cases#	NA	15/23 (65%)	14/19 (74%)	16/21 (76%)	NS
del(13q14)(*D13S25*)#	NA	15/23 (65%)	12/19 (63%)	12/21 (57%)	NS
Trisomy 12#	NA	0/23 (0%)	2/19 (5%)	3/20 (15%)	NS
del(11q)(*ATM*)#	NA	0/23 (0%)	1/19 (5%)	0/20 (0%)	NS
del(17p13)(*TP53*)#	NA	0/23 (0%)	0/19 (0%)	1/20 (5%)	NS
t(14q32) #	NA	1/8 (13%)	1/4 (25%)	0/1 (0%)	NS
% of altered cells/case	NA	17 (5-100)	58 (12-97)	81 (13-99)	<0.03[d,e]

Results expressed as median (range) or as # number of cases (percentage). [a] Controls vs. MBL^{lo}, [b] Controls vs. MBL^{hi}, [c] Controls vs. CLL, [d] MBL^{lo} vs. MBL^{hi}, [e] MBL^{lo} vs. CLL [f] MBL^{hi} vs. CLL. Abbreviations (alphabetical order): **CLL:** chronic lymphocytic leukemia; **M:** mutated *IGHV*; **MBL^{hi}**: high-count monoclonal B-cell lymphocytosis; **MBL^{lo}**: low-count monoclonal B-cell lymphocytosis; **N:** Number; **NA:** Not applicable; **NS:** statistically not significantly different (p>0.05); **UM:** unmutated *IGHV*.

Supplementary figures

SUPPLEMENTARY FIGURE 1. Relative distribution of distinct PB B-cell populations including the subsets of memory B-cells and plasma cells expressing distinct surface immunoglobulin heavy chain (sIgH)-isotype subclasses in four representative subjects from the non-MBL controls, MBL^{lo}, MBL^{hi} and CLL stage 0 study groups.

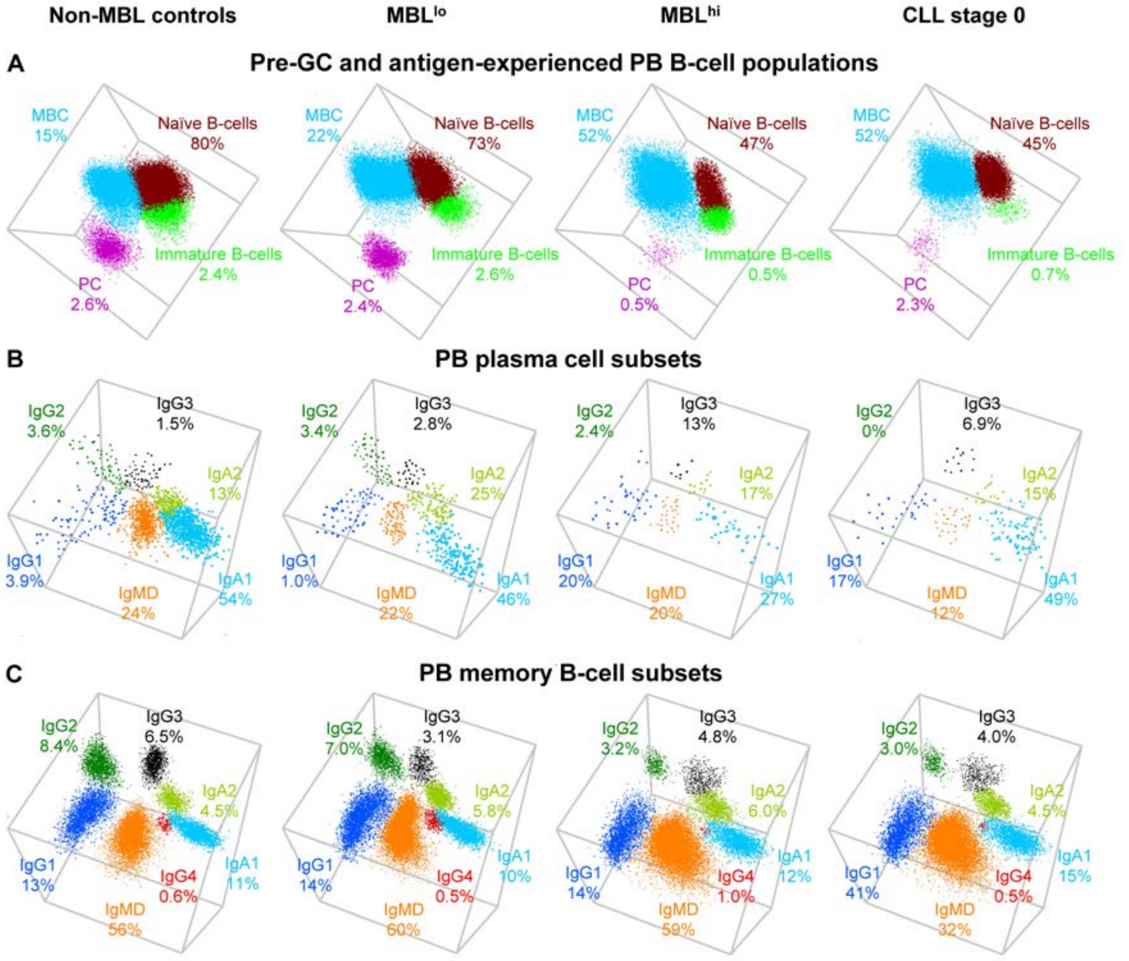

Panel A depicts the relative distribution (from total normal PB B-cells) of pre-germinal center (i.e. immature and naïve B-cells) vs. antigen-experienced B-cells (i.e. memory B-cells and plasma cells) for each of the four cases. **Panels B and C** show the relative (percent) distribution (from total PB memory B cells and total PB plasma cells) of the subsets of memory B-cells and plasma cells expressing different IgH isotypes and subclasses for each case, respectively. Each plot depicts 3-dimensional automated population separator (APS) view -Principal Component 1 (PC1) *vs.* PC2 *vs.* PC3- dot plots obtained from a single representative case within each group of subjects included in this study. **MBL^{lo}:** low-count monoclonal B-cell lymphocytosis; **MBL^{hi}:** high-count monoclonal B-cell lymphocytosis; **CLL:** chronic lymphocytic leukemia; **MBC:** memory B-cells; **PC:** plasma cells. Color codes are identified in each plot.

SUPPLEMENTARY FIGURE 2. Correlation between age and the absolute number of the major total B-cells, memory B-cells and plasma cells and their isotype subclass subsets, as well as the soluble Ig plasma levels in MBL^{lo}, MBL^{hi}, CLL and non-MBL controls subjects.

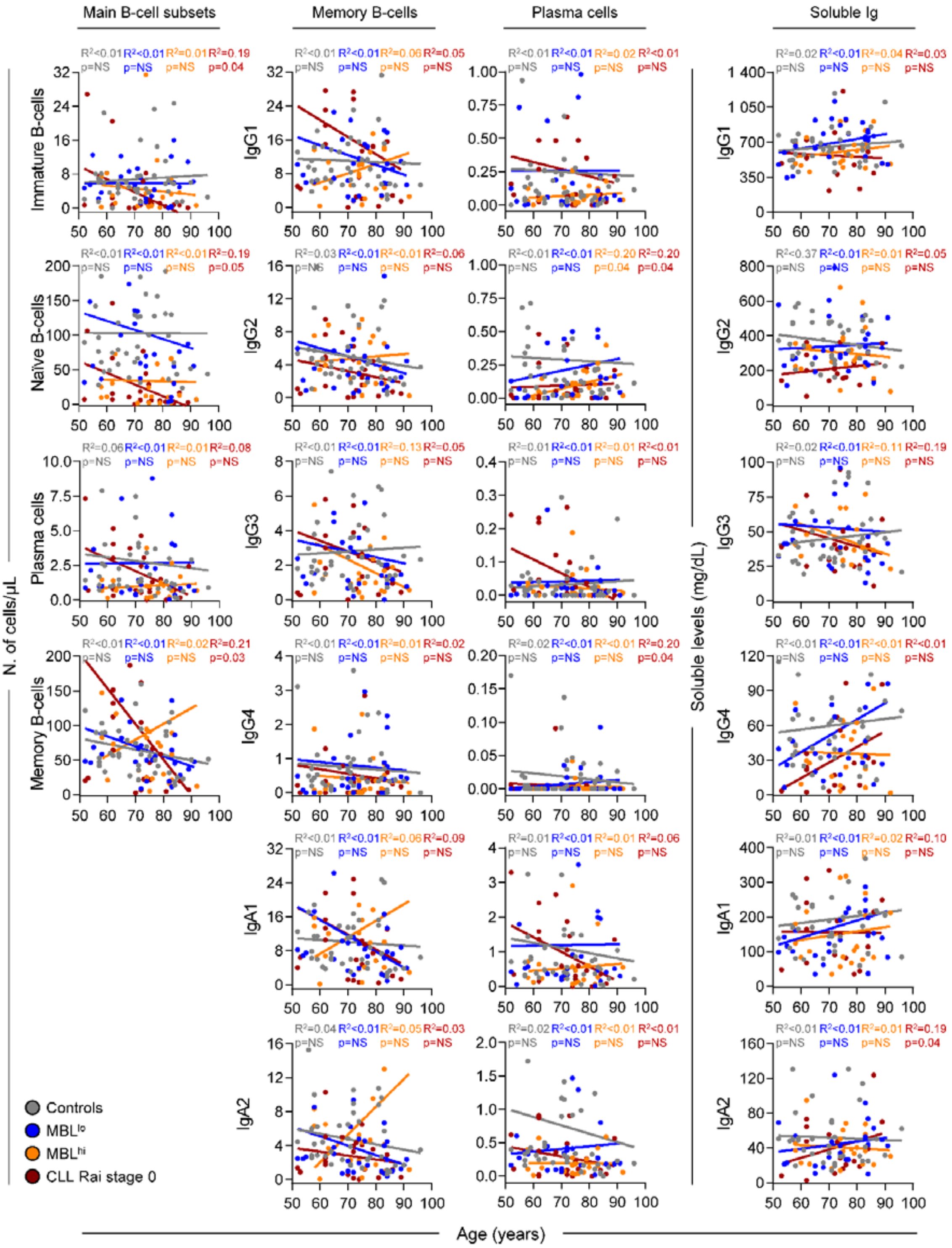

Data plotted in each diagram depicts the Pearson's lineal correlation coefficient (R^2) and its significance (p-value) for age vs. the absolute number of each B-cell subset as well as age vs. Ig soluble plasma levels for individuals within each study group. Color codes as follows: **non-MBL control, gray**; **MBL^{lo}, blue**; **MBL^{hi}, orange**; and **CLL Rai stage 0, red**.

SUPPLEMENTARY FIGURE 3. Distribution of plasma cell subsets expressing different surface immunoglobulin heavy chain (sIgH) isotype-subclasses in peripheral blood of MBL and CLL cases vs. non-MBL controls.

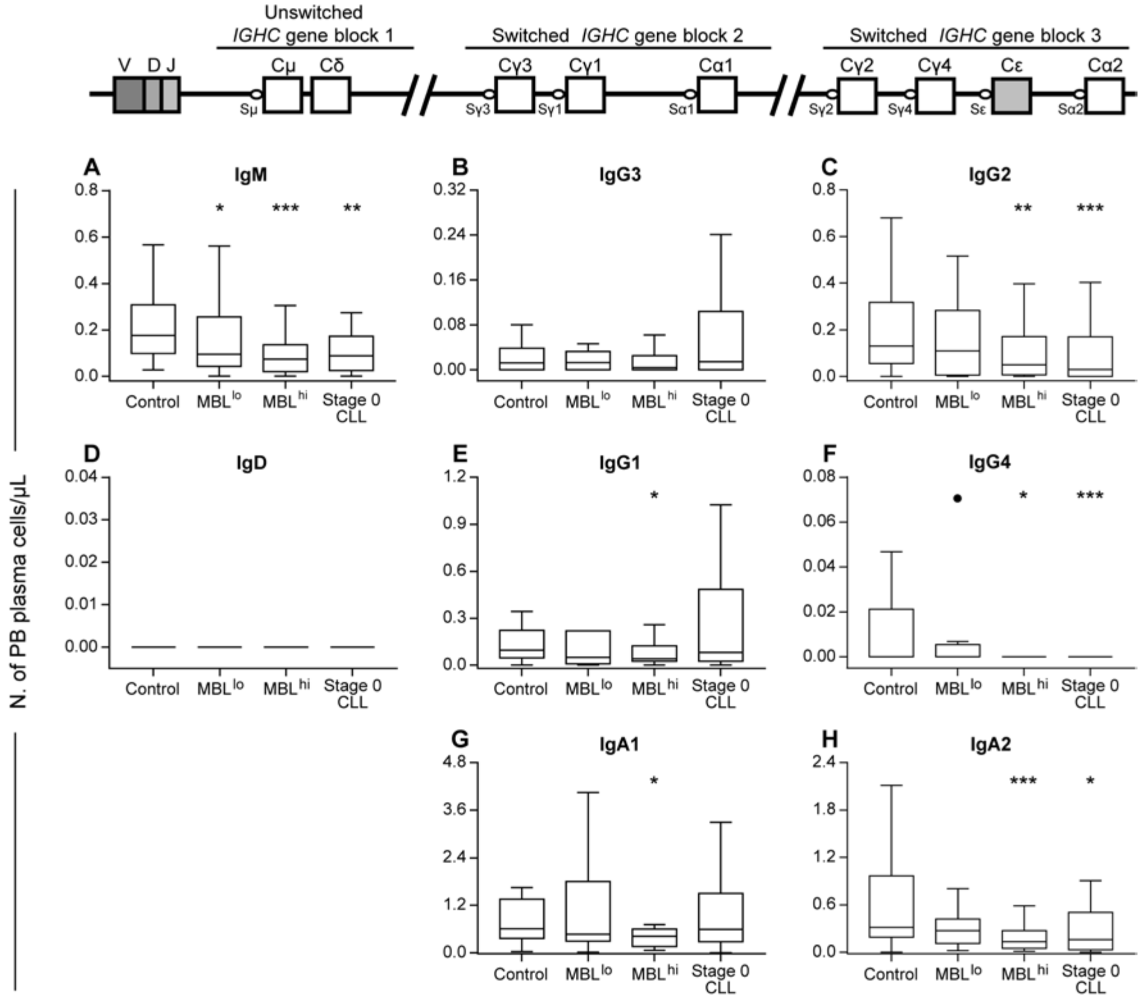

Panels A and D show the distribution in absolute numbers of un-switched IgM$^+$ and IgD$^+$ plasma cells, respectively. **Panels B, E and G** show the absolute number of IgG3$^+$, IgG1$^+$ and IgA1$^+$ switched plasma cells, respectively. **Panels C, F and H** depict the distribution in absolute numbers of IgG2$^+$, IgG4$^+$ and IgA2$^+$ plasma cells, respectively. The relative position and order of gene segments of the *IGHC* gene that encode for the different Ig-subclasses are depicted in the top of the figure. Notched boxes represent 25th and 75th percentile values; the lines in the middle correspond to median values (50th percentile) and vertical lines represent the highest and lowest values that are neither outliers nor extreme values. *P≤0.05 vs. controls; **P≤0.01 vs. controls; ***P≤0.001 vs. controls and $^{\bullet}P$<0.08 vs. controls. **MBL^{lo}:** low-count monoclonal B-cell lymphocytosis; **MBL^{hi}:** high-count monoclonal B-cell lymphocytosis; **CLL:** chronic lymphocytic leukemia.

SUPPLEMENTARY FIGURE 4: Distribution of memory B-cell subsets expressing different surface immunoglobulin heavy chain (sIgH) isotype subclasses in peripheral blood of MBL and CLL cases vs. non-MBL controls.

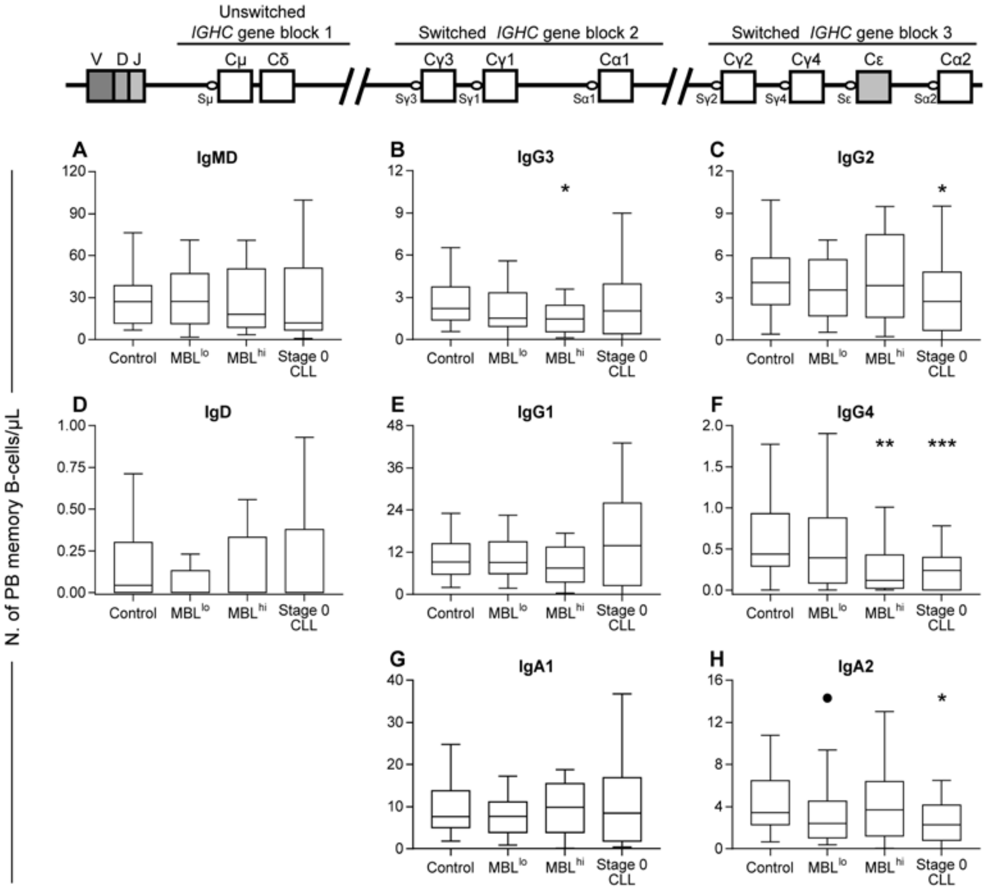

Panels A and D show the distribution in absolute numbers of un-switched $IgMD^+$ and IgD-only memory B-cells, respectively. **Panels B, E and G** depict the absolute numbers of $IgG3^+$, $IgG1^+$ and $IgA1^+$ switched memory B-cells, respectively. **Panels C, F and H** depict the distribution in absolute numbers of $IgG2^+$, $IgG4^+$ and $IgA2^+$ memory B-cells, respectively. The relative position and order of gene segments of the *IGHC* gene that encode for the different Ig-subclasses are depicted in the top of the figure. Notched boxes represent 25th and 75th percentile values; the lines in the middle correspond to median values (50th percentile) and vertical lines represent the highest and lowest values that are neither outliers nor extreme values. **P*≤0.05 vs. controls; ***P*≤0.01 vs. controls; ****P*≤0.001 vs. controls and •*P*<0.08 vs. controls. **MBL^{lo}:** low-count monoclonal B-cell lymphocytosis; **MBL^{hi}:** high-count monoclonal B-cell lymphocytosis; **CLL:** chronic lymphocytic leukemia.

SUPPLEMENTARY FIGURE 5. Distribution of soluble plasma levels of the main Ig isotype subclasses in MBL^{lo}, MBL^{hi} and CLL cases vs. non-MBL healthy subjects (controls).

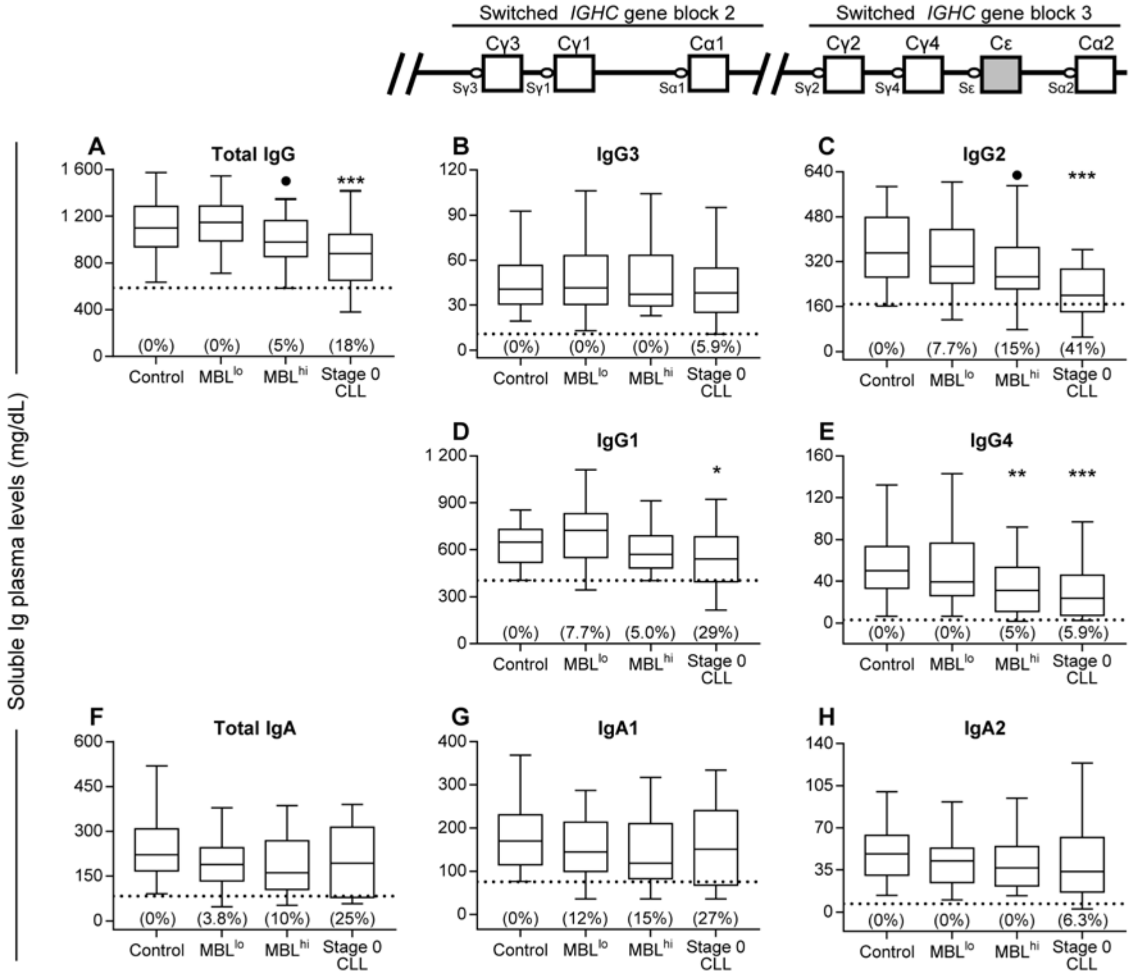

Panel A shows the titer of total plasma IgG. **Panels B, C, D and E** represent the plasma titers of soluble IgG3, IgG2 and IgG1 and IgG4 subclasses, respectively. In turn, **Panel F** displays the plasma levels of total IgA, while **Panels G and H** show the plasma titers of IgA1 and IgA2 subclasses, respectively. Dotted lines correspond to the lower range of normality for each Ig-subclasses, and percentages depict the frequency of cases below the normal range. The relative position and order of gene segments of the *IGHC* gene that encode for the different Ig-subclasses are depicted in the top of the figure. **P*≤0.05 vs. controls; ***P*≤0.01 vs. controls; ****P*≤0.001 vs. controls and $^{\bullet}$*P*<0.08 vs. controls. **MBL^{lo}:** low-count monoclonal B-cell lymphocytosis; **MBL^{hi}:** high-count monoclonal B-cell lymphocytosis; **CLL:** chronic lymphocytic leukemia.

SUPPLEMENTARY REFERENCES:

1 Swerdlow SH, Campo E, Harris NL, Jaffe ES, Pileri SA, Stein H *et al. WHO Classification of Tumours of Haematopoietic and Lymphoid Tissues*. 4th ed. IARC: Lyon (France), 2017.

2 Hallek M, Cheson BD, Catovsky D, Caligaris-Cappio F, Dighiero G, Dohner H *et al.* Guidelines for diagnosis, indications for treatment, response assessment and supportive management of chronic lymphocytic leukemia. *Blood* 2018, e-pub ahead of print 14 March 2018; doi:10.1182/blood-2017-09-806398.

3 Kalina T, Flores-Montero J, van der Velden VHJ, Martin-Ayuso M, Böttcher S, Ritgen M *et al.* EuroFlow standardization of flow cytometer instrument settings and immunophenotyping protocols. *Leukemia* 2012; **26**: 1986–2010.

4 Flores-Montero J, Sanoja-Flores L, Paiva B, Puig N, García-Sánchez O, Böttcher S *et al.* Next Generation Flow for highly sensitive and standardized detection of minimal residual disease in multiple myeloma. *Leukemia* 2017; **31**: 2094–2103.

5 Blanco E, Perez-Andres M, Sanoja-Flores L, Wentink M, Pelak O, Martín-Ayuso M *et al.* Selection and validation of antibody clones against IgG and IgA subclasses in switched memory B-cells and plasma cells. *J Immunol Methods* 2017, e-pub ahead of print 28 September 2017; doi:10.1016/j.jim.2017.09.008.

6 Perez-Andres M, Paiva B, Nieto WG, Caraux A, Schmitz A, Almeida J *et al.* Human peripheral blood B-cell compartments: a crossroad in B-cell traffic. *Cytometry B Clin Cytom* 2010; **78 Suppl 1**: 47-60.

7 Caraux A, Klein B, Paiva B, Bret C, Schmitz A, Fuhler GM *et al.* Circulating human b and plasma cells. age-associated changes in counts and detailed characterization of circulating normal CD138- and CD138 plasma cells. *Haematologica* 2010; **95**: 1016–1020.

8 Quijano S, López A, Rasillo A, Sayagués JM, Barrena S, Sánchez ML *et al.* Impact of trisomy 12, del(13q), del(17p), and del(11q) on the immunophenotype, DNA ploidy status, and proliferative rate of leukemic B-cells in chronic lymphocytic leukemia. *Cytom Part B - Clin Cytom* 2008; **74**: 139–149.

9 van Dongen JJM, Langerak AW, Bruggemann M, Evans PAS, Hummel M, Lavender FL *et al.* Design and standardization of PCR primers and protocols for detection of clonal immunoglobulin and T-cell receptor gene recombinations in suspect lymphoproliferations: report of the BIOMED-2 Concerted Action BMH4-CT98-3936. *Leukemia* 2003; **17**: 2257–2317.

10 Rodríguez-Caballero A, Henriques A, Criado I, Langerak AW, Matarraz S, López A *et al.* Subjects with chronic lymphocytic leukaemia-like B-cell clones with stereotyped B-cell receptors frequently show MDS-associated phenotypes on myeloid cells. *Br J Haematol* 2015; **168**: 258–267.

11 Damle RN, Wasil T, Fais F, Ghiotto F, Valetto A, Allen SL *et al.* Ig V gene mutation status and CD38 expression as novel prognostic indicators in chronic lymphocytic leukemia. *Blood* 1999; **94**: 1840–1847.

12 Hamblin TJ, Davis Z, Gardiner A, Oscier DG, Stevenson FK. Unmutated Ig V(H) genes are associated with a more aggressive form of chronic lymphocytic leukemia. *Blood* 1999; **94**: 1848–1854.

13 Sims GP, Ettinger R, Shirota Y, Yarboro CH, Illei GG, Lipsky PE. Identification and characterization of circulating human transitional B cells. *Blood* 2005; **105**: 4390–4398.

14 Agrawal S, Smith SABC, Tangye SG, Sewell WA. Transitional B cell subsets in human bone marrow. *Clin Exp Immunol* 2013; **174**: 53–59.

15 Blanco E, Perez-Andres M, Arriba-Mendez S, Contreras-Sanfeliciano T, Criado I, Pelak O *et al.* Age-associated distribution of normal B-cell and plasma cell subsets in peripheral blood. *J Allergy Clin Immunol* 2018, e-pub ahead of print 2 March 2018; doi:10.1016/j.jaci.2018.02.017.
